# Biomarkers for Early Stages of Johne’s Disease Infection and Immunization in Goats

**DOI:** 10.3389/fmicb.2018.02284

**Published:** 2018-09-28

**Authors:** Aubrey Berry, Chia-wei Wu, Amanda J. Venturino, Adel M. Talaat

**Affiliations:** The Laboratory of Bacterial Genomics, Department of Pathobiological Sciences, University of Wisconsin–Madison, Madison, WI, United States

**Keywords:** Johne’s disease, goats, transcription, genetic, biomarkers, pathogenesis

## Abstract

**Background:** *Mycobacterium avium* subsp. *paratuberculosis* (*M. paratuberculosis*) is the causative agent of Johne’s disease, a chronic enteric infection of ruminants. Infection occurs within the first few months of life but remains subclinical for an average of 2–5 years. Current diagnostics to detect early subclinical infections lack diagnostic sensitivity, which hinders disease control resulting in significant economic losses to the dairy industry worldwide. The pathophysiology of early infection with *M. paratuberculosis* is still not well understood and represents a key hurdle toward the development of better diagnostics.

**Methods:** The present study employed a large-scale RNA-Sequencing technology to better understand early stages of *M. paratuberculosis* infection and immunization. Specifically, gene expression profiles of peripheral blood mononuclear cells (PBMCs) from infected or vaccinated goats were compared to controls.

**Results:** When compared to the naïve control goats, we identified a large number of transcripts (*N* = 226, 1018, 1714) that were differentially expressed in the *M. paratuberculosis-*infected goats, goats vaccinated with live attenuated or inactivated vaccines. There were also 1133 differentially expressed (DE) transcripts between vaccinated goats and infected ones. Bioinformatics evaluation of the DE genes indicated the regulation of a large number of genes with immunity and inflammatory functions including IL-18BP, IFN-γ, IL-17A, NOS2, LIPG, and IL-22. Interestingly, a large number of goat genes (*N* = 667) were regulated whether live or inactivated vaccine were used. Some of the regulated genes (e.g., IL-17A, IFN-γ) continued its unique transcriptional profile up to 12 months post-challenge.

**Conclusion:** Overall, transcriptome analysis of infected and/or immunized goats identified potential targets for developing early diagnostics for Johne’s disease and a potential approach to differentiate infected from vaccinated animals. A similar approach could be used to analyze later stages of Johne’s disease or other chronic infections.

## Introduction

Johne’s disease, caused by *Mycobacterium avium* subsp. *paratuberculosis* (*M. paratuberculosis*) is a chronic progressive gastroenteritis of ruminants. Clinical signs of the disease include chronic diarrhea, weight loss, low milk yield, and higher mortality than uninfected herds (Ott et al., 1999; Bush et al., 2006; Singh et al., 2007). Johne’s disease causes severe economic losses to the dairy industry worldwide, with estimated losses of $200-$250 million a year in the US alone (Ott et al., 1999). Additionally, *M. paratuberculosis* has been implicated as a potential factor in Crohn’s disease in humans (Waddell et al., 2016). In ruminants, infection primarily occurs early in life through the ingestion of *M. paratuberculosis* contaminated feces, colostrum, or milk. Although infection mainly begins in neonates, clinical signs do not appear until 2–5 years of age (Tiwari et al., 2006). The pathophysiology of *M. paratuberculosis* persistence and survival for years in the host before clinical signs develop is not well understood. In the subclinical phase of *M. paratuberculosis* infection, a cell-mediated immune response is driven by Th1 cytokines with minimal humoral response elicited (Magombedze et al., 2014). *M. paratuberculosis* is known to be able to survive and replicate within host macrophages by subverting macrophage function through mechanisms such as blocking maturation of phagolysosomes (Souza et al., 2013). Progression from subclinical to clinical disease results in a switch to a predominantly humoral response, driven by Th2 cytokines, which is ineffective at controlling the disease (Magombedze et al., 2014).

Current diagnostic tests, such as fecal culture and ELISA, have poor sensitivity (from 19 to 53%) for detection of the subclinical phase of infection (Sockett et al., 1992; McKenna et al., 2005). During this subclinical phase, the host is intermittently shedding *M. paratuberculosis* in feces, contaminating the environment and transmitting the pathogen to progeny and others in the herd (Whittington and Sergeant, 2001). Being able to detect infection early on, would allow for more effective disease control within herds. Biomarkers are increasingly examined for other chronic diseases such as *M. tuberculosis* (Walzl et al., 2011) and have the ability to serve as a detection tool for different stages of disease. Peripheral blood mononuclear cells (PBMCs) have been shown to be a predictor of infection and inflammatory disease (Marteau et al., 2005). Previous studies investigating the gene expression profiles of PBMCs have shown distinct differences between healthy cattle and those with chronic *M. paratuberculosis* infection (Coussens et al., 2004; Verschoor et al., 2010; David et al., 2014). A study using microarrays to identify gene expression in whole blood from calves 3 months post- infection found several putative biomarkers with roles in the immune response (David et al., 2014). Other studies have focused on cows at 6 months or later times post-infection (PI) (Coussens et al., 2004; Verschoor et al., 2010; David et al., 2014). However, a caveat to these studies is that they all detected subclinically infected cows by use of serum ELISA tests which only become positive later in the course of infection. In the present study, we examined gene expression profiles at a much earlier stage (30 days PI) using an experimental infection model. Understanding differences in gene expression profiles will not only improve understanding of the pathophysiology of disease progression but will also allow the identification of novel targets for earlier diagnosis of the subclinical stage of *M. paratuberculosis* infection.

While several other studies have investigated the PBMC transcriptome of cattle infected with *M. paratuberculosis*, this is the first such analysis in goats. Goats are increasingly used as a small ruminant model for Johne’s disease (Hines et al., 2007, 2014; Shippy et al., 2017) and, therefore, understanding the host’s response to infection and differences among ruminant species is vital. One major complication of our preliminary transcriptome analysis of goats (vs. cows) lied in the lack of a fully annotated genome. The ∼2.66-Gb genome draft sequence of the goat (*Capra hircus*) was released in 2013 and was highly fragmented (Dong et al., 2013; Du et al., 2014). Annotation of the goat genome has been performed through GLEAN and, while many genes have been linked to the *Bos taurus* genome through ENSEMBL, many gaps in annotation remain (Lin et al., 2015). A combination of analyses from multiple sequencing platforms and scaffolding technologies significantly improved the assembly (Bickhart et al., 2017). The annotation is still highly dependent on the bovine genome and requires more studies such as transcriptomic analysis to improve. In this report, PBMC’s were collected throughout an ongoing Johne’s disease vaccine trial in goats (Shippy et al., 2017). RNA-Sequencing (RNA-Seq) was used to profile the PBMC transcriptomes of goats at 30 days PI or post-vaccination. Our results provided valuable information on differential gene expression in goats during the early subclinical stage of infection and the host response to vaccination by either a live-attenuated vaccine (LAV) or an inactivated vaccine (Mycopar^®^). These results can be used to identify potential transcripts as early diagnostic biomarkers of infection and to differentiate vaccinated from *M. paratuberculosis*-infected animals.

## Materials and Methods

### Animals

Approximately 1 week-old kids were purchased from a farm with no previous history of Johne’s disease. All study kids, and their dams, tested negative for *M. paratuberculosis* by ELISA for serum antibody (Paracheck^®^, Biocor Animal Health, Omaha, NE, United States). Additionally, fecal samples collected from the originating farm environment were negative for *M. paratuberculosis* by culture. All kids were housed in a restricted biosafety animal facility (BSL-2). All animal care was handled in accordance to the standards of the University of Wisconsin-Madison Animal Care and Use Committee. The kids were randomly assigned to one of four groups as shown in **Table 1**. One group of kids (*n* = 6 but only 4 used for transcriptome analysis) were vaccinated with a LAV construct [*M. paratuberculosis*Δ*lipN* mutant (Wu et al., 2007)] at a dose of 1 × 10^9^ CFU/animal. The second groups of kids (*n* = 4) were vaccinated with the USDA-licensed inactivated vaccine (Mycopar^®^). A third group inoculated with PBS served as the vaccine control. Both vaccines and PBS were given subcutaneously. At 60 days post-vaccination, kids in these three groups were inoculated with *M. paratuberculosis* strain JTC1285 at a dose of 1 × 10^8^ CFU administered orally in the milk replacer for three consecutive days. A fourth group (*n* = 4), inoculated with PBS and not challenged with *M. paratuberculosis* served as a naïve control. Power analysis was used to determine group sizes (Chow et al., 2008) based on a pilot study by our group. Goat kids were monitored daily for signs of clinical disease and evaluated monthly for potential weight loss. A detailed report on the outcome of this vaccine/challenge study was previously published (Shippy et al., 2017).

**Table 1:** Experimental groups used to collect RNA samples for transcriptome analysis.

Group	No.	Vaccine^∗^	Vaccine dose	Challenge strain/Dose^∗∗^
Infected	4	PBS	0.5 ml	*M. paratuberculosis* JTC1285/
				1 × 10^8^ CFU
LAV-	4	*M. ap*	1 × 10^9^ CFU	*M. paratuberculosis* JTC1285/
vaccinated		Δ*lipN*		1 × 10^8^ CFU
Mycopar-	3	Mycopar	0.5 ml	*M. paratuberculosis*
vaccinated				JTC1285/1 × 10^8^ CFU
Naïve	4	PBS	0.5 ml	None
Control				

*^∗^All vaccines were given subcutaneously. ^∗∗^Challenge dose was given orally in milk replacer for three consecutive days and was performed at 60 days post-vaccination in LAV- and Mycopar- vaccinated groups.*

### Isolation of Blood Cells

Blood samples (10 ml) were collected from the jugular vein of goats into EDTA vacutainer tubes pre-vaccination, 1 week, 30 days, 60 days post-vaccination and 1 week post-challenge (for 3 groups), and then monthly for 12 months. Peripheral blood mononuclear cells (PBMC) were isolated using Histopaque^®^-1077 (Sigma–Aldrich^®^) with the following modifications. Anti-coagulated blood was diluted with an equal volume of RPMI-1640 medium (Sigma Aldrich^®^), layered over 10 ml of Histopaque^®^-1077, and centrifuged at 400 ×*g* for 30 min at room temperature. Following centrifugation, PBMC’s were aspirated from the interface and washed twice with RPMI-1640 medium. Residual red blood cells were lysed with 0.83% NH_4_Cl_2_. The PBMC’s were then resuspended in complete culture medium (RPMI-1640 containing 10% fetal bovine serum, 1% L-glutamine, 1% penicillin/streptomycin (final concentration 100 IU/ml), and 1% non-essential amino acids). Cell density was determined by use of 0.4% Trypan blue stain and a hemocytometer.

### PBMC Stimulation and RNA Extraction

Peripheral blood mononuclear cells were plated at a density of 1 × 10^6^/well in 96 well plates with either medium alone (non-stimulated) or *M. paratuberculosis* whole cell lysate (WCL). The WCL was prepared by resuspending the centrifuged cell pellet of actively grown *M. paratuberculosis* (O.D. ∼1.0) in protein lysis buffer (100 mM Tris–Cl, 100 mM NaCl, 5 mM MgCl_2_, 1 mM PMSF, complete ultra-protease inhibitor cocktail (Roche, Indianapolis, IN, United States; pH 7.5) and bead-beating to homogenize (maximum pulse for 45 s for a total of 4 pulses; with cooling on ice for 30 s between pulses). The supernatant was then transferred to a new 1.5 ml tube and non-soluble material was removed by centrifugation at 10,000 ×*g* for 5 min at 4°C. The protein content of the supernatant was measured via the Pierce^TM^ BCA protein assay (Thermo Fisher Scientific), aliquoted and stored at -80°C until used. Final concentrations of WCL was 10 μg/ml. IL-2 was added to all wells at a concentration of 100 U/ml. Plates were incubated at 37°C with 5% CO_2_ for 24 h. Supernatants were then removed and cell pellets were stored in 100 μl TRIzol^®^ and frozen at -80°C until used for RNA extraction. RNA was extracted from stimulated PBMC’s using TRIzol^®^ and RNeasy^®^ Mini Kit (Qiagen^®^) according to manufacturer’s directions for the remainder of the extraction. TURBO DNA-free^TM^ DNase Treatment (Ambion^®^) was used to eliminate residual genomic DNA. RNA quantity and quality was assessed using the RNA Pico Series Chip on the Bioanalyzer 2100 (Agilent). RNA integrity numbers (RINs) > 8 were obtained for all total RNA samples purified.

### RNA Sequence Analysis

RNA-Sequencing (RNA-Seq) was performed by the University of Wisconsin–Madison Biotechnology Center on RNA extracted from WCL-stimulated PBMC’s from goats at 30 days post-vaccination, 30 days post-challenge (PBS vaccinated), or at the same time for the naïve control group (4 goats/biological replicates per group). A total of 1 μg of RNA was used as input for TruSeq RNA Sample Prep Rev.F (March 2014; Illumina). Paired-end RNA Sequencing was performed on the Illumina HiSeq 2000 sequencer according to manufacturer’s instructions.

Raw RNA-Seq reads were uploaded to CLC Genomics Workbench 8.5 (Qiagen, Redwood City, CA, United States) for processing. Two read files from one RNA sample were paired and trimmed. The ambiguous trim limit was set at 1 and quality trim limit was at 0.05. Reads shorter than 25 nucleotides were excluded. The trimmed sequences were then mapped to the reference genome sequence of *Capra hircus* assembly ARS1 (Bickhart et al., 2017) and read counts against the reference genome annotation tracks, generated with files, available at ftp://ftp.ncbi.nlm.nih.gov/genomes/Capra_hircus, were compiled and tabulated using the CLC Genomics Workbench NGS tools. The mapping parameters were set as follows: mismatch cost, 2; insertion and deletion cost, 3; length and similarity fraction, 0.8. Unique gene reads from each sample were exported from CLC Genomics Workbench and used for normalization and differential gene expression analysis with an R package, DESeq2 version 1.16.1 (Love et al., 2014). Transcripts that had an average of normalized read count < 3 in all three tested groups were excluded from the analysis (*N* = 11,541). Differentially expressed (DE) transcripts are defined as transcripts with fold changes ≥ 2.0 or ≤-2.0 (or Log_2_-transformed fold changes ≥ 1.0 or ≤-1.0), and *p*-value < 0.05 when compared to the naïve control group.

Gene ontology (GO) analysis was performed for the DE genes with agriGO, an automated tool to identify enriched GO terms, which is specially focused on agricultural species (Du et al., 2010). The gene products are categorized with respect to biological processes, cellular components, and molecular functions. Because the GO in the goat genome is poorly annotated, we chose the *Bos taurus* ENSEMBL genome B2G list (2010 version) as the reference genome. Goat genes (assembly ARS1) with an Entrez gene name were mapped to the counterparts in the bovine genome, resulting in a total gene list of 9,115 GO-annotated genes. Goat DE genes identified in the RNA-Seq analysis were also mapped to the bovine genome and used as query lists against the 9,115-gene reference. FDR was calculated using the Fisher test.

Network analysis was performed using the STRING database (Szklarczyk et al., 2015) with DE transcripts identified in this study. The input DE transcripts were treated as homologues of *Bos taurus* because of availability in the database.

### Quantitative RT-PCR

cDNA was synthesized from each RNA sample using SuperScript III Reverse Transcriptase (Invitrogen, Waltham, MA, United States) and oligo(dT)_12-18_ Primer according to manufacturer’s instructions. Quantitative PCR (qPCR) assays were performed in triplicates for each cDNA sample. Primers were designed across adjacent exons in order to differentiate products from genomic DNA and cDNA (**Supplementary Table S9**). The GAPDH gene served as an internal control to normalize the data for the ΔΔ*Ct* relative quantitation method. The assays were performed on an Applied Biosystems StepOne Plus Real-Time PCR System (Foster City, CA, United States), and the cDNA amplifications were monitored by the measurement of SYBR Green fluorescence at a specific cycle threshold. Each reaction was carried out in a 20 μl volume that contained 10 μl of 2× GoTaq qPCR Master Mix (Promega, Madison, WI, United States), 5.0 μl of ddH2O, 0.5 μl of each primer (10 μm) and 4.0 μl of the template (100–150 ng/ul). The qPCR amplification process began with the temperature at 95°C for 2 min, followed by 40 cycles of the amplification process (95°C for 3 s, 60°C for 30 s). Subsequent to the cycling process, melting curves were generated by inclining the temperature from 60°C to 95°C at 0.3°C/s increments. With the exception of the infected group at 1 month post-challenge where two samples were used, cDNA samples from three animals in each group were included in the qPCR analysis. Average ΔΔ*Ct* values and standard errors of the mean (SEM) of the three measurements were calculated and transformed to linear fold change.

## Results

### Transcriptome Analysis of Goat Groups

The transcriptome analysis of goats infected with *M. paratuberculosis* and/or vaccinated LAV vaccine strain *M. ap*Δ*lipN* is a proportion of a larger study that examined the performance of this vaccine published earlier (Shippy et al., 2017). The transcriptome analysis is the focus of this report. The summary statistics of the RNA-Seq data for each replicate are shown in **Table 2**. Mean values of 58.88 million raw reads were generated per library (each RNA sample). Following trimming of reads based on read length, quality score and adapter sequences, an average of 20.04 million paired reads remained. Alignment of the trimmed RNA-Seq reads to the *Capra hircus* reference genome yielded mean values per library of 18.71 million paired reads (93.32%) mapped to unique locations.

**Table 2:** Summary statistics for Illumina RNA sequencing data from individual animals.

Group/Replicate number^∗^	Total number of reads trimmed	Number of read pairs being mapping	Total paired reads after mapping	% Total paired reads after trimming	Uniquely mapped reads	% Uniquely mapped reads
Infected 1	73,105,634	22,810,292	25,147,671	68.8	23,533,313	93.58
Infected 2	72,155,108	23,491,132	24,331,988	67.44	22,701,378	93.30
Infected 3	68,005,382	21,295,316	23,355,033	68.69	21,941,420	93.95
Infected 4	48,108,726	15,062,194	16,523,266	68.69	15,433,817	93.41
LAV-vaccinated 1	73,973,058	24,360,882	24,806,088	67.07	23,253,486	93.74
LAV-vaccinated 2	63,076,126	20,765,850	21,155,138	67.08	19,668,167	92.97
LAV-vaccinated 3	34,967,370	11,169,558	11,898,906	68.06	11,062,093	92.97
LAV-vaccinated 4	66,996,260	21,387,972	22,804,144	68.08	21,282,727	93.33
Mycopar-vaccinated 1	60,074,726	20,141,682	19,966,522	66.47	18,707,059	93.69
Mycopar-vaccinated 2	70,284,036	23,645,792	23,319,122	66.36	21,978,596	94.25
Mycopar-vaccinated 3	64,746,832	21,633,920	21,556,456	66.59	20,141,993	93.44
Naive 1	73,575,076	22,859,182	25,357,947	68.93	23,681,558	93.39
Naive 2	50,851,250	16,107,620	17,371,815	68.32	16,180,977	93.14
Naive 3	46,913,012	15,083,746	15,914,633	67.85	14,815,443	93.09
Naive 4	34,829,124	11,209,870	11,809,627	67.81	10,972,712	92.91

*^∗^Time when blood samples were taken: Infected: 30 days post-infection; LAV-vaccinated: 30 days post-LAV vaccination; Mycopar-vaccinated: 30 days post-Mycopar vaccination; Naïve:: 30 days post-PBS vaccination.*

### Changes in the Goat Transcriptomes Related to Infection or Vaccination

**FIGURE 1:**
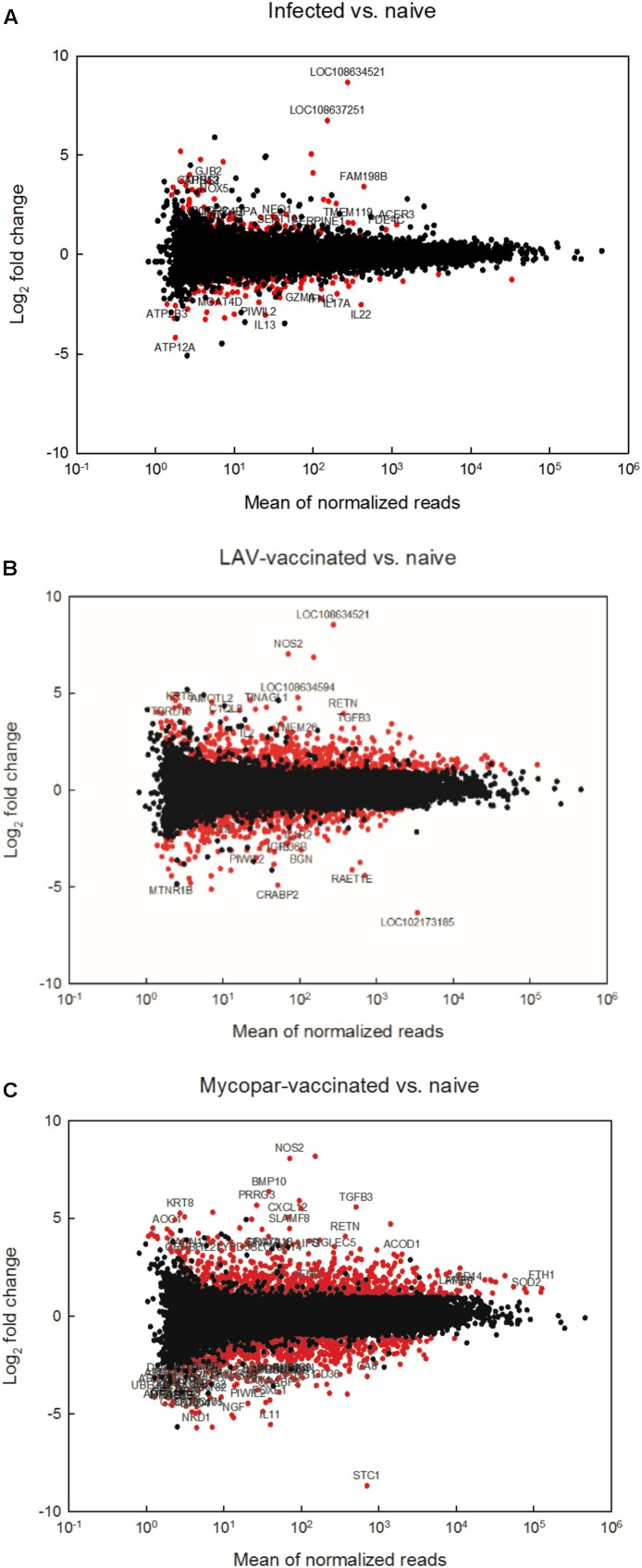
RNA sequencing analysis of different goat groups following infection or vaccination with LAV or Mycopar^®^ vaccines. MAplots of **(A)** the infected group compared to the naïve group, **(B)** the LAV-vaccinated group compared to the naïve group and **(C)** the Mycopar-vaccinated group compared to the naïve group are shown. Red dots represent differentially expressed transcripts (fold change > 2.0 or <–2.0, *p* < 0.05).

Transcriptomes of different animal groups were analyzed to identify DE genes with significant change using a *p*-value threshold of ≥ 0.05 and ≥ 2-fold change (**Supplementary Table S1**). A summary of comparative numbers of DE genes is presented in **Table 3**. MA-plots in **Figure 1** depict the distributions of the DE transcripts PI and post-vaccination groups compared to naïve control group. Generally, the infected goat group had 226 significantly DE transcripts out of 17,380 (total goat transcripts identified by RNA-Seq) at 30 days PI in comparison to the naïve, non-infected controls. Of the 226 significantly DE transcripts, 113 were up-regulated in the PI group, while the other 113 were down-regulated. A total of 106 out of the 226 DE transcripts had more than a 2.8-fold change (or 1.5 log_2_ fold change) with a selected group of known function listed in **Table 4**. On the other hand, the LAV-vaccinated goat group had 1018 significantly DE transcripts out of 17,380 compared to the naïve, non-infected control group. A total of 628 and 390 transcripts were up- and down-regulated, respectively. A total of 517 out of the 1018 DE transcripts had ≥2.8 fold change with a selected group of known function listed in **Table 5**. Additionally, when the transcripts of both LAV-vaccinated and infected groups were compared, at total of 1133 transcripts were significantly DE out of 17,380 (**Table 3**). Of these transcripts, 629 and 504 transcripts were up- and down-regulated, respectively. A total of 575 out of the 1133 DE transcripts were greater than a 2.8 fold change. Interestingly, the immunization with the inactivated, oil-based vaccine (Mycopar) triggered significant changes in a large number of goat genes (*N* = 1714) including key genes involved in immune responses (**Table 6**).

**Table 3:** Differentially expressed (DE) genes for each comparison group.

Comparison	Total analyzed Genes	DE genes^∗^
Infected vs. Naïve	17,380	226
LAV-vaccinated vs. Naïve		1,018
Mycopar-vaccinated vs. Naïve		1,714
LAV-vaccinated vs. Infected		1,133

*^∗^DE genes were identified as those with a p-value threshold of <0.05.*

**Table 4:** Selected differentially up- or down-regulated genes by fold change, between 30 days post-infection and naïve groups among 226 genes with significant differential expression.

Gene symbol	Gene ID	Fold change	*P*-value	Description
FAM198B	102191727	10.70	0.0016	Family with sequence similarity 198 member B
CDCP1	102187276	4.06	0.0143	CUB domain containing protein 1
TMTC1	102185637	3.63	0.0217	Transmembrane and tetratricopeptide repeat containing 1
BAIAP2L1	102173150	3.61	0.0196	BAI1 associated protein 2 like 1
MEI1	102169168	2.99	0.0155	Meiotic double-stranded break formation protein 1
SETP10	102171885	2.41	0.0239	Septin 10
IL17F	102171111	-2.33	0.0098	Interleukin 17F
FCER2	102171507	-2.57	0.0001	Fc fragment of IgE receptor II
IFNG	100860815	-3.36	0.0047	Interferon, gamma
ADGRG1	102171366	-3.43	0.0037	Adhesion G protein-coupled receptor G1
APBB1	102179305	-4.47	0.0002	Amyloid beta precursor protein binding family B member 1
PIWIL2	102173845	-5.31	0.0400	Piwi like RNA-mediated gene silencing 2

**Table 5:** Selected differentially up- or down-regulated genes by fold change, between 30 days post-LAV-vaccination and naïve groups among 1018 genes with significant differential expression.

Gene symbol	Gene ID	Fold change	*p*-value	Description
NOS2	100860742	130.42	2.3E-09	Nitric oxide synthase 2
TINAGL1	102169636	19.31	1.2E-05	Tubulointerstitial nephritis antigen like
RETN	102176742	12.91	4.4E-13	Resistin
C1QL2	102176742	12.89	0.002	Complement C1q like 2
TDRD10	102174259	11.54	0.019	Tudor domain containing 10
TGFB3	102189962	9.13	0.0020	Transforming growth factor beta 3
ADGRE2	102171592	5.90	0.0135	Adhesion G protein-coupled receptor E2
LIPG	102191574	5.28	0.0001	Lipase G, endothelial type
KCNJ2	102168940	4.82	0.0003	Potassium voltage-gated channel subfamily J member 2
AQP9	102181396	4.72	0.0007	Aquaporin 9
BPI	102185756	3.61	0.0140	Bactericidal/permeability-increasing, protein
IL9	102179848	-2.91	0.0083	Interleukin 9
IL1R2	102186601	-3.63	0.0055	Interleukin 1 receptor type 2
IL36B	102182235	-5.46	0.0013	Interleukin 36 beta
IGF1	100860838	-5.46	0.0463	Insulin, like, growth, factor, 1
BGN	102183219	-8.57	0.0045	Biglycan
PIWIL2	102173845	-8.57	0.009	Piwi like RNA-mediated gene silencing 2
RAET1E	108636743	-17.27	0.0008	Retinoic acid early transcript 1E
CRABP2	102174348	-30.12	2.0E-20	Cellular retinoic acid binding protein 2

**Table 6:** Selected differentially up- or down-regulated genes by fold change, between 30 days post-Mycopar-vaccination and naïve groups among 1714 genes with significant differential expression.

Gene symbol	Gene ID	Fold change	*p*-value	Description
NOS2	100860742	269.200	3.7E-11	Nitric oxide synthase 2
BMP10	102185577	82.746	0.0003	Bone morphogenetic protein 10
TDRD10	102174259	18.438	0.0061	Tudor domain containing 10
RETN	102170965	16.901	4.2E-12	Resistin
AMOTL2	102169708	14.389	0.0065	Angiomotin like 2
KLRG2	102177407	12.733	2.9E-10	Killer cell lectin like receptor G2
IL21	100861248	8.124	4.6E-05	Interleukin 21
C2	102176085	7.95	2.4E-7	Complement C2
C3	100860826	6.495	0.0002	Complement C3
MCEMP1	102172348	6.436	7.4E-08	Mast cell expressed membrane protein 1
IL34	102173115	5.434	0.0084	Interleukin 34
IL12A	100861293	3.907	0.0035	Interleukin 12A
TLR4	100860955	3.423	3.8E-07	Toll like receptor 4
TNF	100861232	3.399	0.0003	Tumor necrosis factor
IL18	100861190	-4.441	3.6E-06	Interleukin 18
IL9	102179848	-4.802	0.0012	Interleukin 9
IL9R	102191479	-4.961	9.4E-08	Interleukin 9 receptor
IL5	102188034	-4.964	0.0396	Interleukin 5
IL36B	102182235	-9.557	0.0001	Interleukin 36 beta
IL13	102187477	-9.675	3.4E-07	Interleukin 13
PIWIL2	102173845	-22.152	0.0009	Piwi like RNA-mediated gene silencing 2
IL11	102184367	-46.823	1.6E-07	Interleukin 11

Several genes involved in immune responses were significantly regulated in all goat groups. For example, leukemia inhibitory factor (LIF), interferon-gamma (IFN-γ), and interleukin 22 (IL-22), were found to be DE genes in the infected group when compared to both the control and the LAV-vaccinated groups. More gene lists are provided in **Supplementary Tables S2–S8**. In the infected group, LIF was down-regulated by -2.51 fold change when compared to the control group and by 3.84 fold when compared to the vaccinated group. IL-22, a Th17-related cytokine, was also down-regulated by a -5.78 fold in the infected group vs. the control group and by -33.82 fold when compared to the LAV-vaccinated group. Interestingly, NOS2 gene involved in controlling infection of a closely related mycobacteria, *M. tuberculosis* (Kutsch et al., 1999; Velez et al., 2009), was significantly induced (>100 fold) in both vaccine groups, suggesting an important role of this gene in adaptive immune responses following immunization with LAV (**Table 5**) or inactivated (**Table 6**) vaccine. A group of genes with unique diphasic regulatory responses in both LAV and infected goats included immune response genes (e.g., IFN-γ, Granulysin) as well as basic cell metabolic process (e.g., ART5). This list of genes (**Table 7**) could expand gene categories utilized as targets for developing a sensitive assay to differentiate infected from vaccinated animals (DIVA).

**Table 7:** Common differentially expressed genes regulated in opposite direction between 30 days post-infection and 30 days post-LAV-vaccinated groups, each compared to the naïve group.

Gene symbol	Gene ID	Fold change in infected group	Fold change in vaccinated group	Description
LOC106503226	106503226	2.62	-2.53	Non-coding RNA
PMP22	102184371	2.11	-3.46	Peripheral myelin protein 22
ART5	102169686	-2.01	3.05	ADP-ribosyltransferase 5
LOC102169116	102169116	-2.03	2.27	Ecto-ADP-ribosyltransferase 5
GNLY	102191341	-2.13	2.19	Granulysin
ASAP3	102182646	-2.16	2.10	ArfGAP with SH3 domain ankyrin repeat and PH domain 3
LOC108633178	108633178	-2.68	2.95	Granzyme B-like
TBKBP1	102172659	-3.03	2.23	TBK1 binding protein transcript
SLC17A7	102169042	-3.12	5.50	Solute carrier family 17 member 7
LOC108638192	108638192	-3.27	5.28	Non-coding RNA
IFNG	100860815	-3.36	3.89	Interferon gamma

**FIGURE 2:**
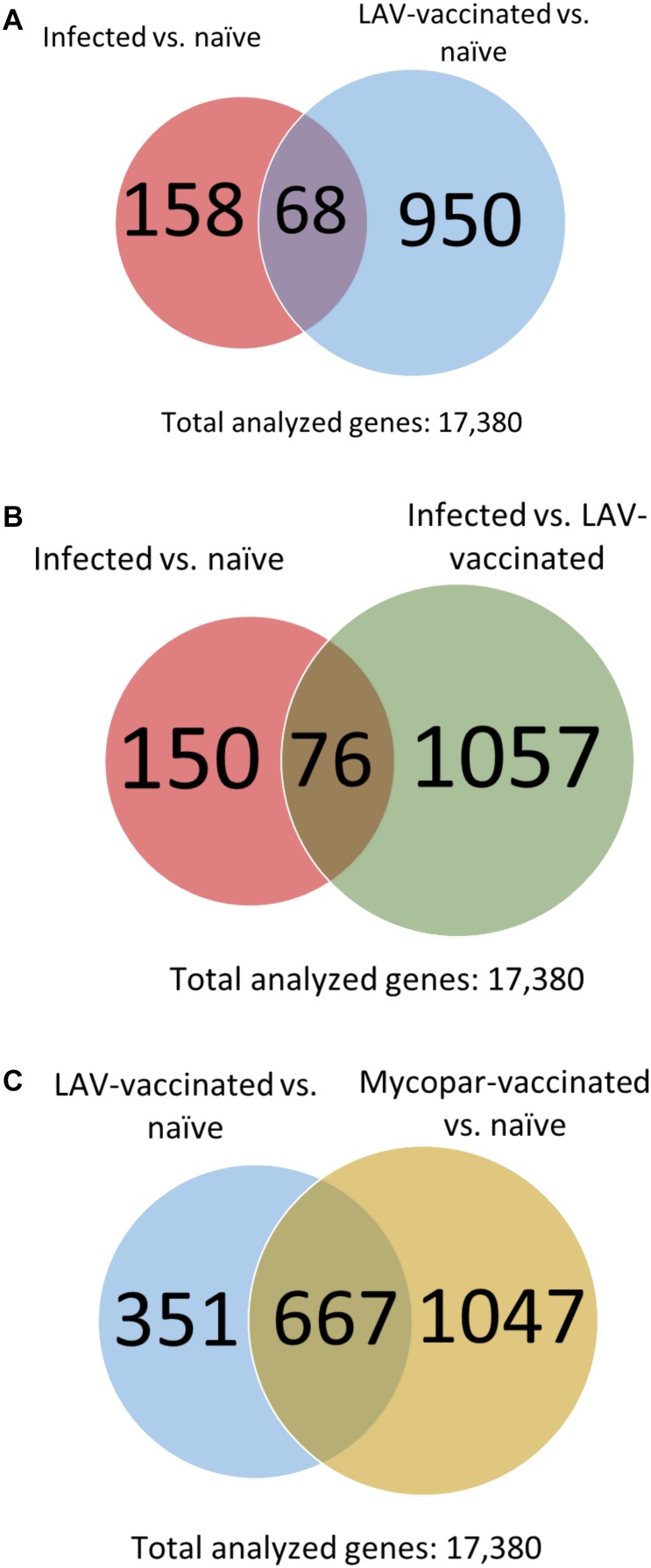
Comparative transcriptome analysis of the infected and vaccinated goat groups. Venn diagrams show numbers of common DE transcripts between **(A)** the infected and vaccinated groups, each compared to the naïve group. **(B)** Infected vs. naïve and infected vs. LAV-vaccinated groups and **(C)** LAV-vaccinated vs. naïve and Mycopar-vaccinated vs. naïve groups.

Among those identified DE transcripts in the infected and LAV-vaccinated groups (each referenced against the naïve group), there were 68 transcripts in common (**Figure 2A**). The majority of those transcripts were regulated in the same direction in both groups, but 11 transcripts were regulated in the opposite direction. A non-coding RNA transcript, LOC106503226 and a gene, PMP22, were the only two that were up-regulated 30 days PI and down-regulated 30 days post-vaccination. The remaining 9 transcripts (e.g., ART5 and IFNG) were down-regulated 30 days PI and up-regulated 30 days post-vaccination (**Table 6**). More comparative analysis of transcript profiles identified 76 transcripts commonly up- or down-regulated shared between the lists of genes from comparing infected vs. naïve control and *M. paratuberculosis*-infected vs. LAV-vaccinated transcripts (**Figure 2B**). Those common genes could be considered the core responsive genes for *M. paratuberculosis* infection or vaccination with an LAV vaccine. For the inactivated vaccine, a total of 667 core genes (**Figure 2C**) were also regulated when compared to the LAV-vaccine group (**Supplementary Table S7**). Such core genes included those with potential rules in immunity (e.g., NOS2, RETN, and IL21), another indication of core genes responsive to any *M. paratuberculosis-*specific vaccines whether live-attenuated or inactivated were used.

### Pathways and Networks of Differentially Expressed Genes

**FIGURE 3:**
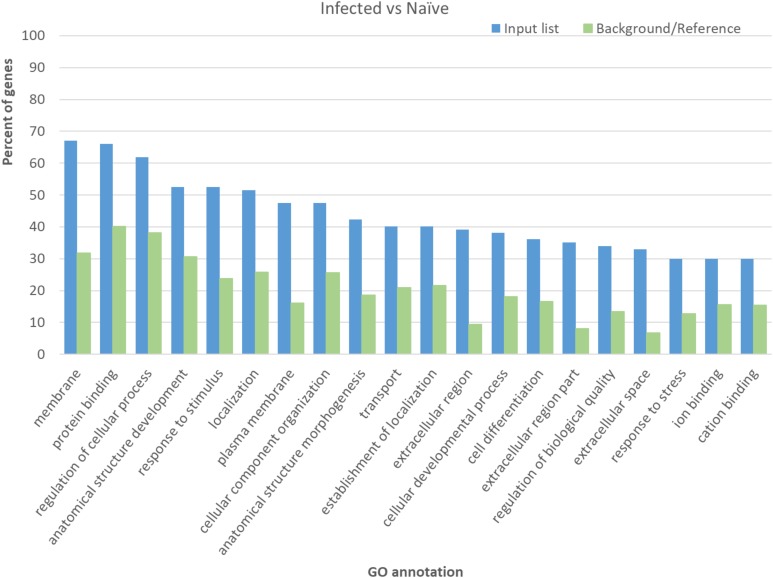
Significant terms in gene ontology analysis for the differentially expressed genes in infected goats compared to naïve control goats. The agriGO for automated identification of GO terms were used on the list of genes with significant differential expression when the transcriptomes of infected and naïve goats were compared.

To better define gene pathways involved in *M. paratuberculosis* infection, genes with significant differential expression were evaluated through GO analysis using agriGO. This analysis provides categories of genes involved in different biological or molecular functions and those integral for different cellular components. Interestingly, the most abundant significant terms for the GO analysis for the infected vs. naïve control group included genes involved in protein binding, regulation of cellular process and response to stimulus, which includes significant subcategories immune responses (GO:0006955) and inflammatory response (GO:0006954) (**Figure 3**), suggesting the importance of controlling immune genes by *M. paratuberculosis* following infection. On the other hand, the largest gene groups with significant GO terms for the Mycopar- or LAV-vaccinated vs. infected groups included genes involved in binding, cellular process and metabolic process while those for the LAV-vaccinated vs. infected group included genes involved in cellular process and biological regulation (**Supplementary Figure S1**).

**FIGURE 4:**
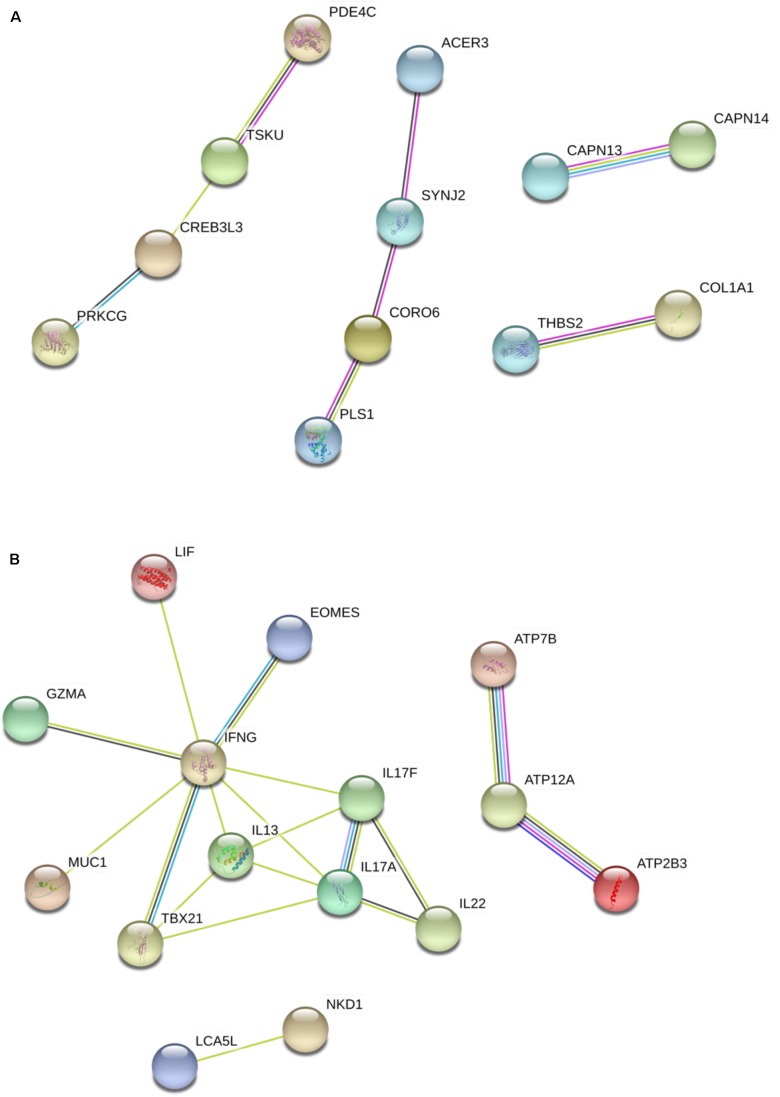
Gene network analysis of DE genes in the infected group. **(A)** Genes that were significantly up-regulated. **(B)** Genes that were significantly down-regulated. IL-17A and IL-17F are two homologs in the IL-17 family. Light green lines represent connection between genes co-mentioned in an abstract in published studies, cyan lines represent putative pathway connections found in homologs in other species, black lines represent co-expression in *Bos taurus* or homologs in other species and pink lines represent experimentally determined association.

To better characterize gene networks activated during infection and vaccination, gene transcripts were further analyzed to identify co-regulated genes. **Figure 4** displays gene network analysis in the post-infection group. Several in the up-regulated group of genes (**Figure 4A**), such as ACER3, SYNJ2, CORO6 and PLS1, showed physical associations and co-expression among transcripts of the *M. paratuberculosis*-infected group. In addition, homologs of PDE4C and TSKU were also found associated (Halls and Cooper, 2010; Schlecht et al., 2012; Costanzo et al., 2016) and suggested to be involved in signaling and relaxin regulation (Halls and Cooper, 2010). In **Figure 4B**, a co-down-regulation of ATP7B, ATP12A and ATP2B3 suggests a possible reduced activity of calcium transport in infected cells. This analysis also highlighted the negative regulation by *M. paratuberculosis* of host cytokines such as IFN-γ, IL-13, IL-17A, IL-17F, and IL-22.

### Prolonged Changes of Key Host Genes

**FIGURE 5:**
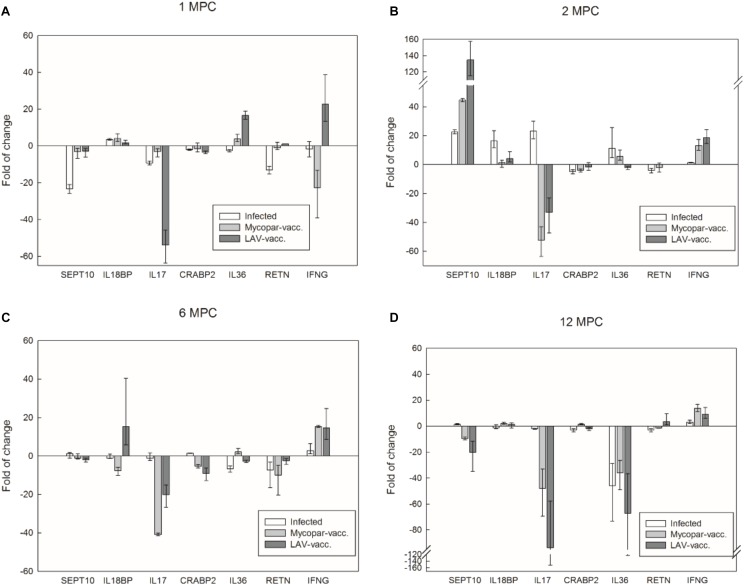
Transcriptional profile of key caprine genes over 12 months post challenge with *M. paratuberculosis*. Panels display quantitative real-time PCR analysis of total RNA extracted from blood samples collected from goat groups at 1 **(A)**, 2 **(B)**, 6 **(C)**, and 12 **(D)** months post challenge (MPC). Expression levels were calculated with ΔΔ*Ct* relative quantitation method relative to the GAPDH gene expression in the naïve group. Target gene names are listed below each panel and fold change for the infected, Mycopar or LAV-vaccinated relative to naïve goat groups are listed on the *Y*-axis. At each time point, samples from three animals in each group except the infected group at 1 MPC (*N* = 2) were included and standard errors of the mean (SEM) of the three measurements were presented as error bars.

To further analyze the utility of transcriptome analysis for prediction of unique transcripts associated with infection or vaccination, we used real-time, quantitative PCR to compare transcript levels among animal groups over 12 months post-challenge (MPC) (**Figure 5**). Interestingly, IL-17 cytokine was repressed in the challenged and Mycopar^®^ and LAV-vaccinated goats compared to the naïve control group for all examined times, except for the infected group at 2 MPC. Similarly, the Sept10 gene was induced, only at 2 MPC. On the other hand, IL-36 was activated soon after vaccination (1 and 2 MPC) but then repressed for the rest of the examined time points, i.e., 6 and 12 MPC. More interestingly, the IFN-γ expression profile was refractive to elicited immune responses. IFN-γ was induced soon in the LAV-vaccine group (1 MPC) but then continued to be expressed in the Mycopar^®^-vaccinated and *M. paratuberculosis*-challenged groups starting from 2 MPC until the end of the experiment. At all of these sampling times, the IFN-γ was consistently higher in the LAV-vaccine group compared to the challenged group.

## Discussion

Infection with *M. paratuberculosis* is costing the dairy industry significant economic losses (Cho et al., 2012) and is difficult to detect its presence, especially during early disease stages (Li et al., 2017). In this project, the goat PBMC transcriptome was profiled using RNA-Sequencing (RNA-Seq) to compare the early gene expression, 30 days post-infection and post-vaccination, compared to healthy, naïve controls. In addition to better understanding of disease progression, such analysis is expected to yield targets for further development into a diagnostic assay for early stages of Johne’s disease. Similar to others (Aranday-Cortes et al., 2012; Bhuju et al., 2012; Churbanov and Milligan, 2012; Kohler et al., 2015), we focused our analysis on the transcriptome of PBMC stimulated cells with *M. paratuberculosis* lysate to further increase biomarker specificity to *M. paratuberculosis* infection and/or vaccination. Many transcriptomic analyzing tools largely depend on information from an annotated genome. In this study, our quality of transcriptomic analyses improved as the goat genome assembly was significantly refined (Bickhart et al., 2017). According to NCBI *Capra hircus* Annotation Release 102, of 20,593 predicted coding genes, 20,256 had a protein aligned 50% or more of the query against the UniProtKB/Swiss-Prot curated proteins (NCBI, 2016). The updated annotation thus provides a much more reliable reference to our analysis. The generated RNA-Seq dataset could also benefit further improvement of goat genome annotation. As expected, a large number of DE transcripts were found between the vaccinated and infected groups (1133 genes) and between the vaccinated and naïve control group (1018 genes). In contrast, there was a relatively small number (226) of DE transcripts when comparing the infected and naïve control group. This large difference in the number of DE transcripts is most likely associated with the route of administration since both vaccines were administered subcutaneously (contrary to oral infection), allowing for increased contact with PBMCs in the bloodstream, while challenge dose of *M. paratuberculosis* could reach PBMC following intestinal invasion (Stabel et al., 2009). Our analysis, further illustrated the importance of route of infection and/or vaccination for the type and magnitude of the generated host responses.

Although the comparison between the infected and naïve control group produced a relatively small number of DE transcripts, preliminary evaluation of these genes indicated a large number of genes with immunological and inflammatory functions, including interferon gamma (IFN-γ), IL-18 binding protein, IL-17A, and IL-22. IFN-γ is an important player in the defense against intracellular pathogens including mycobacteria (Arsenault et al., 2012). A previous study in cattle showed that in the subclinical stages of infection, IFN-γ expression increased at the site of infection (Sweeney et al., 1998). Other studies indicate that *M. paratuberculosis*-infected animals produce IFN-γ but are unresponsive to it (Arsenault et al., 2012). In that study, IFN-γ was secreted significantly less (-3.36 fold change) in subclinically infected goats compared with the naïve, control goats. This IFN-γ profile was also evident in subclinically infected goats vs. vaccinated goats (-13.0 fold change). Previously, IFN-γ was reported to be induced in PBMC’s stimulated with *M. paratuberculosis* whole-cell sonicate from subclinically infected cows (Stabel, 2000). However, these cows ranged from 2 to 10 years of age and therefore were much further along in the infection pathogenesis than in the current study, which tested goats 30 days PI. The host response clearly changes over time and this data may demonstrate that. Potentially linked to the identified repression of IFN-γ, is the moderate up-regulation (+1.30 fold change) of interleukin 18 binding protein (IL-18 bp) in the infected vs. naïve control group. IL-18 bp binds to IL-18 to block its biological activity (Novick et al., 1999). IL-18 is a pro-inflammatory cytokine that functions in the early Th1 cytokine response and induces IFN-γ production. A major source of IL-18 bp is from intestinal endothelial cells and macrophages (Corbaz et al., 2002). Therefore, IL-18 bp serves to modulate the early Th1 immune response in the intestine, the site of *M. paratuberculosis* infection. Interestingly, IL-18 bp has been found to be up-regulated during active Crohn’s disease, an inflammatory bowel disease in humans with potential association to *M. paratuberculosis* infection (Corbaz et al., 2002).

As expected, genes involved in immune responses (e.g., LIF, IFN-γ, and IL-22), were found to be DE among examined goat groups. LIF is a pleiotropic cytokine belonging to the IL-6 cytokine family with receptors primarily on monocytes/macrophages (Nicola and Babon, 2015). In the infected group, both LIF and IL-22, a Th17-related cytokine, were down-regulated in the infected group vs. the control or the vaccinated groups. These three genes, along with IL-13 and IL-17, were also found having associations in the protein network analysis. IL-17 was also down-regulated in the infected vs. control group. Down-regulation of IFN-γ, IL-22 and IL-17 genes may suggest overall down-regulation of Th1 and Th17 cell activities and reduced cellular immunity against infections. Several studies in *Mycobacterium tuberculosis* and *Mycobacterium bovis* have shown significant IL-17 responses (Blanco et al., 2011; Jurado et al., 2012). A recent study on RNA-Seq analysis in cattle infected with *M. bovis* showed an up-regulation of IL-17, IL-22, and IFN-γ at 1-month PI (Waters et al., 2015). This is in contrast to some of our findings in the present study (in case of IL-17) which was further confirmed by prolonged analysis of key genes up to 12 months post infection (**Figure 5**). Such difference could be attributed to the difference in host response to *M. bovis* vs. *M. paratuberculosis.* Further investigation into these key immune regulated genes as will aid in understanding how the host is dynamically responding to *M. paratuberculosis* infection or vaccination.

Our gene network analysis also shows associations among genes that were up-regulated in the infected group (**Figure 4A**). Interestingly, homologs of ACER3, SYNJ2, CORO6 and PLS1 in animal species other than goats (mainly bovine, *Bos Taurus*) were also shown to have physical associations (Schlecht et al., 2012; Hein et al., 2015) and co-expression (Clancy et al., 2003; Janji et al., 2010) as well. Particularly, homologs of CORO6, an actin binding protein, was suggested to be involved in cytokinesis. In *M. tuberculosis*-infected macrophages, CORO6 homolog coronin-1a was suggested to inhibit auto-phagosome formation and facilitate *M. tuberculosis* survival (Seto et al., 2012). In addition, homologs of PDE4C and TSKU were also found associated (Halls and Cooper, 2010; Schlecht et al., 2012; Costanzo et al., 2016) and suggested to be involved in signaling and relaxin regulation (Halls and Cooper, 2010). It may thus imply a status of progression of an *M. paratuberculosis* infection in hosts as observed in *M. tuberculosis* infection (Seto et al., 2012). This observation, along with the likely reduced cellular immunity discussed above, is consistent with the infection status of the host. It is unclear, however, how bacterial or host factors regulate the expression of those genes. Understanding the host-pathogen interaction early in infection will allow for the identification of genes upregulated during initial infection. A useful biomarker for infection must be specific, detectable over the course of the disease with varying inoculation doses, and easily measurable. Moreover, it would improve interpretation of early disease detection if the biomarkers could differentiate infected and vaccinated animals. In our analyses, we identified 9 transcripts (out of 11 in **Table 7**) that were down-regulated 30 days PI and up-regulated 30 days post-vaccination. This biphasic regulation of those genes or transcripts might make them specific markers for differentiating vaccinated animals that are healthy or those infected with *M. paratuberculosis*.

The RNA-Seq analysis was performed only on samples taken 1 month post-infection or post-vaccination to identify early gene regulations in tested groups, notably, between 1 month after vaccinated only and infected only groups. This comparison differentiates host gene regulating responses after exposure to vaccine strains or virulent strains of *M. paratuberculosis*. The vaccinated animals were then challenged 2 months after the vaccination and several key gene expressions were profiled with quantitative PCR (**Figure 5**). The temporal expression patterns within the tested 1 year period could reflect unique characteristics of host responses after exposure to virulent *M. paratuberculosis* with or without prior vaccinations and could also benefit development of diagnostics. For example, IL-17 expressions in the vaccinated animals remained highly repressed at all time while peaking at 2 month post-challenge in the infected only group.

Future work includes evaluation of these target genes in calves at various time points of infection phase to confirm our analysis of experimentally infected animals. Similarly, we could identify specific biomarkers for the differentiation of infected from vaccinated animals, a goal that could further improve the utility of using vaccines to control Johne’s disease in cattle.

## Data Availability

All data presented in this manuscript are available through this report or the accompanied **Supplementary Tables** and **Supplementary Figures**. RNA-Seq data were deposited in NCBI Gene Expression Omnibus (GEO) database under accession number GSE117799.

## Author Contributions

AT perceived the original idea and supervised the whole project. AB and CWW conducted all of the experiments while AV was responsible for the real-time PCR section. All the authors contributed to the writing and editing of the manuscript.

## Conflict of Interest Statement

AT has an ownership interest in Pan Genome Systems, Inc., which is working in the area of animal vaccine development. The remaining authors declare that the research was conducted in the absence of any commercial or financial relationships that could be construed as a potential conflict of interest.
