# Supplementary material for: Biomarkers for Early Stages of Johne’s Disease Infection and Immunization in Goats
Source: Front Microbiol. 2018 Sep 28;9:2284. doi: 10.3389/fmicb.2018.02284 (PMC6172484; doi:10.3389/fmicb.2018.02284)
Supplement: Supplementary file 2 [file Data_Sheet_1.PDF]

a. Mycopar-vaccinated vs. Naïve group

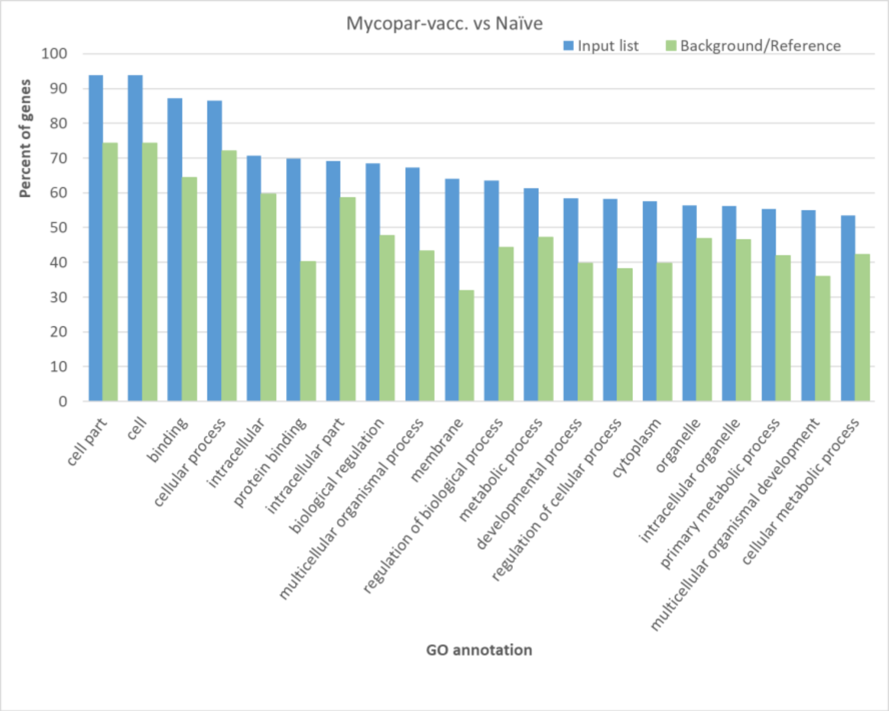

b. LAV-vaccinated vs. Naïve Group

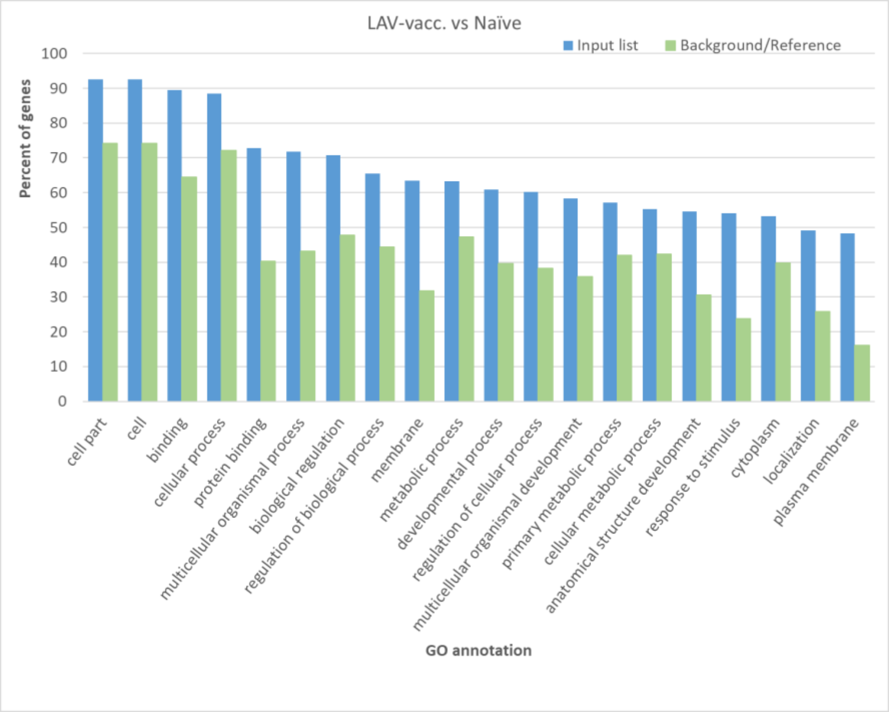

c. LAV-vaccinated vs. Infected group

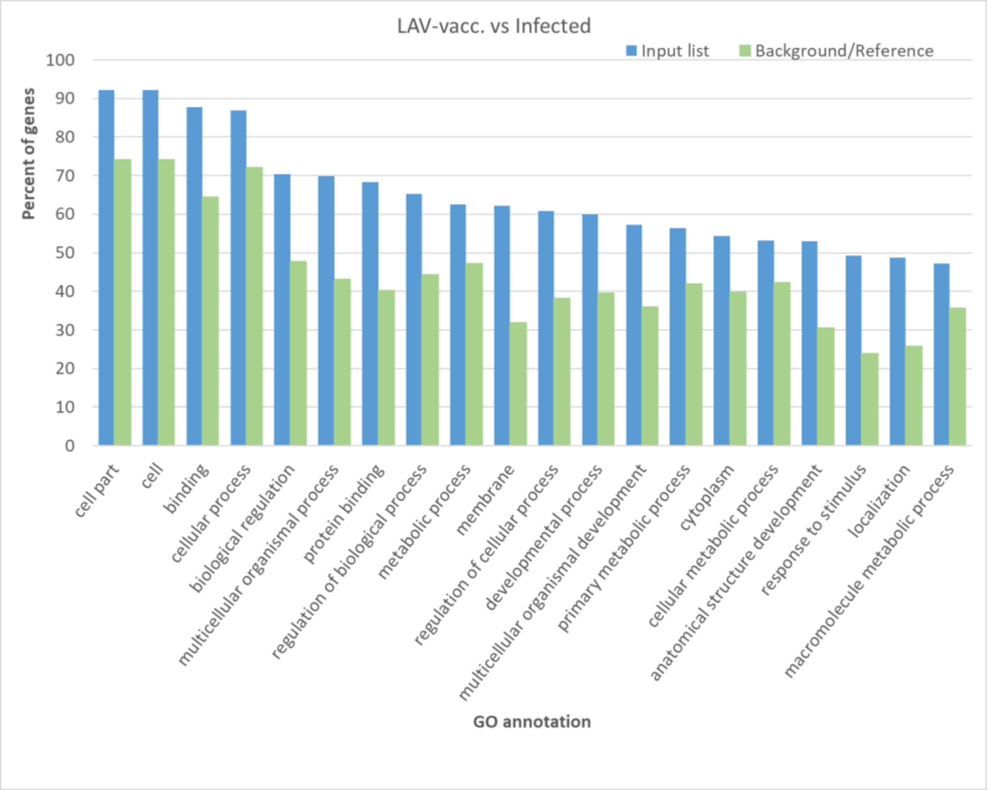

Figure S1. Significant terms in gene ontology analysis, using agriGO, for the differentially expressed genes. The significant terms for the GO analysis for the Mycopar-vaccinated vs naïve group is shown in (a). Chart in (b) shows the significant terms for LAV-vaccinated vs naïve group and in (c) shows the significant terms for LAV-vaccinated vs. infected group. The significant GO terms for both comparisons are 1-binding, 2-intracellular, 3-intracellular part and 4-metabolic process.
